# Curcumin Could Prevent the Development of Chronic Neuropathic Pain in Rats with Peripheral Nerve Injury^☆^

**DOI:** 10.1016/j.curtheres.2012.10.001

**Published:** 2013-06

**Authors:** Younghoon Jeon, Chae-Eun Kim, Dongho Jung, Kyunghwa Kwak, Sungsik Park, Donggun Lim, Sioh Kim, Woonyi Baek

**Affiliations:** 1 Department of Anesthesiology and Pain Medicine, School of Dentistry, Kyungpook National University, Daegu, Republic of Korea; 2 Department of Food Science and Nutrition, Kyungpook National University, Daegu, Republic of Korea; 3 Department of Anesthesiology and Pain Medicine, Kyungpook National University Hospital, Daegu, Republic of Korea

**Keywords:** curcumin, mitogen-activated protein kinases, neuropathic pain

## Abstract

**Background:**

Peripheral nerve injury results in chronic neuropathic pain characterized by allodynia and/or spontaneous pain. It has been suggested that activation of mitogen-activated protein kinases such as extracellular signal-regulated kinase (ERK) and c-Jun N-terminal kinase (JNK) contribute to the neuropathic pain.

**Objectives:**

We investigated if curcumin could prevent the development of neuropathic pain in rats with chronic constriction injury (CCI) of the sciatic nerve.

**Methods:**

The animals were divided into 3 groups. In the curcumin treatment group (n = 10), curcumin (50 mg/kg/d PO) was administered once daily from 1 day before CCI to 7 days after CCI. The rats in the sham group (n = 10) and CCI group (n = 10) received a control vehicle. The mechanical allodynia was assessed using von Frey at 1, 3, 5, and 7 days after nerve injury. Western blots were used to evaluate the levels of p-ERK, p-JNK, and phosphorylation of NR1 (p-NR1) subunits of N-methyl-D-aspartate in the spinal dorsal root ganglion.

**Results:**

In the CCI group, mechanical allodynia was observed during 7 days after nerve injury. However, curcumin treatment reversed the mechanical allodynia 7 days after nerve ligation. There were no differences in the expression of p-ERK, p-JNK, and p-NR1 between the sham and curcumin groups. However, the expression of p-ERK, p-JNK, and p-NR1 in the CCI group were higher than the sham group and curcumin group, respectively (*P* < 0.05).

**Conclusions:**

Treatment with curcumin during the early stages of peripheral neuropathy can prevent the development of chronic neuropathic pain.

## Introduction

Peripheral nerve injury often results in chronic neuropathic pain that is characterized by allodynia and/or spontaneous pain. Treatment of neuropathic pain is a challenge because the underlying mechanisms are not clearly understood and current analgesics, such as anticonvulsant or opiates, are limited in relieving this pain because of their partial efficacy and potential toxicity.^1^

Recently, increasing evidence shows that mitogen-activated protein kinases such as extracellular signal-regulated kinase (ERK) and c-Jun N-terminal kinase (JNK) play important roles in the generation of chronic pain such as neuropathic and inflammatory pain.^2–4^ The expression of phosphorylated ERK (p-ERK) in the spinal dorsal ganglion (DRG) has been implicated in the induction of neuropathic pain behavior in rat models of chronic constriction injury (CCI) and its normalization after decompression of CCI reflects the reversal of the pain behaviors.^5^ The expression of phosphorylated JNK (p-JNK) is also activated in the spinal DRG after nerve injury, and this expression of p-JNK can maintain mechanical allodynia.^6^

Curcumin is a polyphenol found in the dietary spice turmeric (*Curcuma longa* Linn.). It is extracted from dried rhizomes of the perennial herb, which is a member of the ginger family.^7^ Curcumin has been demonstrated to have a variety of biologic activities, including anti-inflammatory activities and anticancer properities.^8,9^ Curcumin is known to exert its action through inhibition of mitogen-activated protein kinases.^10^ Therefore, we investigated if curcumin would prevent the development of neuropathic pain though its inhibitory action on p-ERK and p- JNK in spinal DRG and reduce phosphorylation of NR1 (p-NR1) subunits of N-methyl-D-aspartate in rats with CCI.

## Materials and Methods

### Experiment animals

Thirty adult male Sprague Dawley rats (weighing 280 to 320 g) were used in the experiments. Rats were randomly divided into 3 groups: the sham group (n = 10), CCI group (n = 10), and curcumin group (n = 10). The animals were housed under optimal laboratory conditions, maintained on a natural light and dark cycle (light cycle: 7:00 am to 7:00 pm), and had a free access to food and water ad libitum. All of the animal experiments were performed in accordance with the National Institutes of Health guidelines on animal care.

### CCI procedure

Rats were anesthetized with enflurane in 60% nitric oxide/40% oxygen during surgical procedures. Rats in the CCI and curcumin groups underwent CCI surgery. CCI surgery was carried out as described previously.^11^ The left sciatic nerve was exposed and, proximal to the trifurcation, approximately 7 mm of the common sciatic nerve was freed of adhering tissue. Four ligatures (chromic gut [4-0]) were loosely tied around the nerve at intervals of approximately 1 mm. The wounds were cleaned with saline, closed with wound clips, and rats were returned to their cage after recovering from anesthesia. Sham-operation control rats underwent an identical surgical procedure as the CCI rats except that the left sciatic nerve was without ligation.

### Drug treatment schedule

Curcumin was purchased from Sigma (St. Louis, Missouri). Curcumin suspension was prepared in a 0.5% carboxymethylcellulose solution. Drug suspension was freshly prepared and administered in a constant volume of 1 mL/100 g body weight. Due to poor absorption, increasing the dose of curcumin did not necessarily result in higher absorption. Sixty percent of the given dose of curcumin was absorbed. It was detected in blood from 15 minutes to 24 hours after administration of curcumin.^12^ Treatment with curcumin at varying dose (15 to 60 mg/kg/d PO) attenuated thermal hyperalgesia in a diabetic mouse model of neuropathic pain.^13^ In the curcumin treatment group, therefore, curcumin (50 mg/kg/d PO) was administered from 1 day before CCI surgery to 7 days after CCI surgery. The rats in the control group and CCI group received a control vehicle.

### Behavior test

The experimenter who conducted the behavior test was blinded to the nature of the experimental manipulation to avoid bias. The behavior test for mechanical allodynia was performed between 8:00 pm and 12:00 pm. Tactile allodynia was determined by measuring the paw withdrawal thresholdwithdrawal threshold (PWT) in response to probing with a series of von Frey filaments. To quantify mechanical allodynia, a rat was placed under a transparent plastic box on a metal mesh floor. PWT to a series of calibrated von Frey filaments (Somedic, Hörby, Sweden) were measured according to the up-and-down protocol. The examiner touched the plantar surface of the hindpaw with a selected filament. A positive response was defined as a brisk withdrawal or paw flinching. Five stimuli using the selected filament were applied at 5-second intervals. If there was no withdrawal response to the initially selected filament with these 5 stimuli, a filament with a stronger stimulus was applied. If the animal withdrew its hindpaw in response to any of the 5 stimuli, a filament of the next weaker stimulus was chosen. The mechanical threshold was defined as the minimal force (in grams) initiating a withdrawal response. The measurements of PWT were performed 1 day before CCI and 1, 3, 5, and 7 days following CCI.

### Western blot

After mechanical allodynia were measured on the seventh day following CCI, rats were sacrificed for Western immunoblotting. The L4/5 spinal DRG (ipsilateral side only) were quickly removed, frozen immediately on dry ice, and stored at −70°C until use. At the time of assay, each spinal DRG sample was thawed and homogenized in a lysis buffer (20 mM tris[hydroxymethyl]aminomethane pH 8.0; 150 mM sodium chloride; 1 mM EDTA; 2 mM sodium orthovanadate_;_ 0.5 mM dithiothreitol; 10% glycerol; and 1% octylphenoxypolyethoxyethanol, a nonionic, nondenaturing detergent) containing protease inhibitor cocktail (Roche, Mannheim, Germany). After centrifuging at 12,000 rpm for 20 minutes at 4°C, the supernatant was decanted from the pellet and used for Western blot analyses. The concentration of protein in the homogenate was measured using the Bio-Rad Protein Assay Kit I (Bio-Rad, Hercules, California). The homogenate of equal amounts of protein (50 μg of each protein sample) was boiled for 5 minutes at 100°C in gel loading buffer (0.5 M tris[hydroxymethyl]aminomethane, glycerol, 10% sodium dodecyl sulfate, and 0.5% bromophenol blue), run on 10% sodium dodecyl sulfate-polyacrylamide gels, and then transferred to nitrocellulose membrane at 60 V for 3 hours. The membranes were blocked in 3% to 5% nonfat dried milk in tris buffered saline (50 mM tris pH 7.4 and 10 mM sodium chloride) for 1 hour at room temperature and then incubated overnight at 4°C with the primary antibody against p-ERK 1/2 at a dilution of 1:1000 and p-JNK (Santa Cruz Biotechnology, Santa Cruz, California) and p-NR-1 at a dilution of 1:500 (Upstate Biotechnology, Temecula, California). After washing with tris buffered saline (50 mM tris pH 7.4 and 10 mM sodium chloride), the membranes were incubated for 1 hour at room temperature with peroxidase conjugated secondary antibody (1:2000) and washed again. Proteins were detected using ECL system (Amersham Biosciences, Buckinghamshire, England) and band density was semiquantitatively analyzed with the National Institutes of Health Image program. The blots were exposed to autoradiographic film (Eastman Kodak Co, Rochester, New York), the films were scanned into a computer, and the intensity of immunoreactive bands of interest was quantified using Meta Image series software.

### Statistical analysis

Behavior-related data are presented as mean (SEM) and analyzed using the SAS 8.0 statistical program (SAS Institute Inc, Cary, North Carolina).

The behavior data were analyzed by using repeated measured ANOVA, followed by Tukey's test to investigate differences at different times in each group. The Kruskal–Wallis test was used to determine differences in p-ERK, p-JNK, and p-NR1 among the 3 groups, and post hoc comparisons between groups were made using Dunn’s test. A *P* value < 0.05 was considered statistically significant.

## Results

### Behavior test

**Figure 1:**
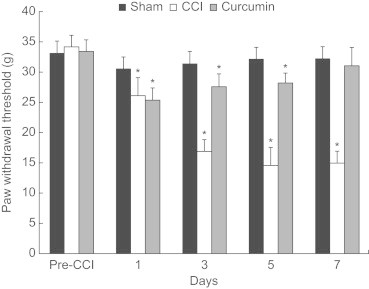
Effects of curcumin (50 mg/kg/d, PO) on mechanical threshold values to von Frey in rats with chronic constriction injury (CCI) of the sciatic nerve. The data are presented as mean (SEM) (n = 10 each group). *P < 0.05 versus pre-CCI.

There were no differences in the mechanical thresholds in the sham group. However, a significant reduction in the mechanical thresholds was observed 1 day after nerve injury (vs pre-CCI; *P* < 0.05), thus indicating development of mechanical allodynia (see Figure 1). In the CCI group, this mechanical allodynia was observed during the 7 days following nerve injury. However, curcumin treatment reversed the mechanical allodynia 7 days after nerve ligation.

### Western blot analysis

**Figure 2:**
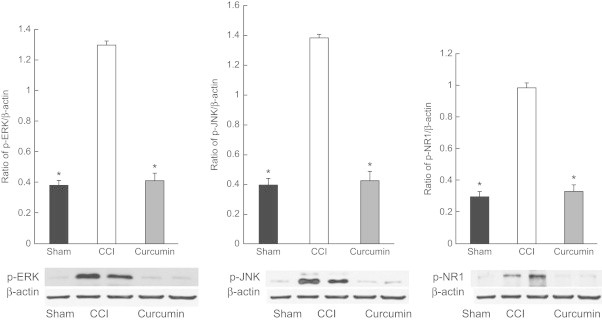
Effects of curcumin (50 mg/kg/d, PO) on phosphorylated extracellular signal-regulated kinase (p-ERK), phosphorylated Jun-N-terminal kinase (p-JNK), and phosphorylation of NR1 (p-NR1) in spinal dorsal root ganglion of rats with chronic constriction injury (CCI) of the sciatic nerve. The data are presented as mean (SEM) (n = 10 each group). *P < 0.05 versus CCI group.

Quantification of Western blots showed that a procedure of nerve ligation resulted in increased levels of p-ERK, p-JNK, and p-NR1 compared with those of the sham group (*P* < 0.05). However, there were no differences in the level of p-ERK, p-JNK, and p-NR1 in the spinal DRG between the sham and curcumin group (*P* > 0.05) (Figure 2).

## Discussion

In our study curcumin reversed the development of mechanical allodynia induced by CCI of the sciatic nerve. It suppresses activation of ERK and JNK in the spinal DRG.

Recently, several studies have reported that ERK may have a role in persistent hyperalgesia (ie, hypersensitivity to thermal and mechanical stimuli).^14,15^ Phosphorylation of ERK may be required for the modulation of nociceptive information produced by intense noxious stimuli and/or peripheral tissue inflammation.^14,15^ Preventing ERK activation with mitogen extracellular kinase inhibitors reduces chronic pain induced by nerve injury.^16^ ERK phosphorylation in the spinal DRG is correlated with neuropathic pain behaviors.^5^ JNK is also rapidly activated in response to environmentally stressful stimuli.^17^ After nerve injury, JNK activation primarily occurs in small diameter C-fiber neurons.^6^ Intrathecal infusion of the peptide JNK inhibitor D-JNKI-1 prevented the development of mechanical allodynia.^6^ In our study, curcumin suppressed phosphorylation of ERK and JNK. In addition, curcumin treatment reversed mechanical allodynia. Recent studies have shown that curcumin is both a nitric oxide scavenger and an inhibitor of inducible nitric oxide synthase expression.^13,18^ Curcumin is also known to reduce the amount of peroxynitrite formed by the reaction between oxygen and nitric oxide and generated from sodium nitroprusside.^19^ Earlier studies have shown that oxidative stress and free radical species are involved in neuropathic pain.^20,21^ Nitric oxide modulates spinal and sensory neuron excitability that contributes to different pain states.^21^ Inducible nitric oxide synthase is involved in the development of hypersensitivity to pain in inflammatory and neuropathic pain models.^22^ From our results, we determine that curcumin has antinociceptive activity, possibly through its inhibitory action on ERK and JNK in neuropathic pain models associated with peripheral nerve injury. However, further studies are warranted to explore the exact mechanism of curcumin's antinociceptive effect.

N-methyl-D-aspartate receptors expressed in spinal DRG have been implicated in the activity-dependent plastic changes that lead to the generation and maintenance of central sensitization, which is believed to be an important mechanism underlying chronic neuropathic pain. ^23,24^ In our study, 7 days after nerve ligation there were no differences in the level of p-NR1 in the spinal DRG between the sham and curcumin groups, whereas the level of p-NR1 in the CCI group significantly increased compared with those of sham group. However, it was probably too early to see the development of central sensitization. Therefore, another study is needed to examine if long-term administration with curcumin prevents central sensitization in rats with chronic neuropathic pain.

## Conclusions

Treatment with curcumin during the early stages of peripheral neuropathy can prevent the development of chronic neuropathic pain.

## Conflicts of Interest

The authors have indicated that they have no conflicts of interest regarding the content of this article.
